# Studies on the Origin of Carbons in Aroma Compounds from [^13^C_6_]Glucose -Cysteine-(E)-2-Nonenal Model Reaction Systems

**DOI:** 10.3390/polym11030521

**Published:** 2019-03-19

**Authors:** Ze Song, Qian Jia, Miaomiao Shi, Tao Feng, Shiqing Song

**Affiliations:** 1 School of Perfume and Aroma Technology, Shanghai Institute of Technology, Shanghai 201418, China; songze_sunny@sina.cn (Z.S.); 176071204@mail.sit.edu.cn (Q.J.); fengtao@sit.edu.cn (T.F.); 2 School of Food and Biological Engineering, Zhengzhou University of Light Industry, Zhengzhou 450002, China; chengzi3090@126.com

**Keywords:** Maillard reaction, carbon module labeling, [^13^C_6_]glucose, cysteine, (E)-2-nonenal

## Abstract

The thermal degradation of lipid oxidation products with amino acids and reducing sugars is known to be important for the characteristic aroma generation in both meat and meat-like process flavorings. SPME(solid phase microextraction)/GC-MS was used to analyze the volatiles produced from a solution of [^13^C_6_]glucose, cysteine, and lipid degradation product- (E)-2-nonenal, heated at 130 °C for 90 min. Analysis of the mass spectra showed that the resulting 2-butyl-thiophene and 5-butyldihydro-2(3H)-furanone were ^13^C_6_-labeled and hence stemmed from glucose. Glucose and (E)-2-nonenal were equally important for the formation of 2-pentylfuran, whether cysteine was present in the reaction or not. 2-Furanmethanol, (E)-2-(1-pentenyl)-furan, 2-hexanoylfuran, ethanethiol, 5-methyl-2(5H)-thiophenone, 1-methyl-5-mercaptotetrazole, 4-pentyl-pyridine, 2-pentyl-thiophene, and 2-mercaptopropanoic acid were virtually ^13^C_1_-^13^C_4_ labeled, suggesting an origin from both glucose and cysteine and/or (E)-2-nonenal carbons. Thus, the relative contribution of aldehyde to the C-skeleton of a particular aroma compound changed substantially when both glucose and cysteine were involved in its formation.

## 1. Introduction

Over the past few years, many studies have been conducted on the production of meat flavor and the interaction between Maillard reactions and lipid degradation during the thermal processing of meat products [1]. Lipid oxidation products have been degraded to lipid aldehydes, acids, and ketones, which may have inherent flavors contributing to the fatty aroma [2]. On the other hand, these degraded products may participate further in Maillard reactions to yield other compounds that contribute to flavor or modify the balance of odorous compounds [3].

Generally, model systems of amino acid/peptide-reducing sugars with lipids have been used to study the influence of lipids on flavor formation mechanisms. For example, Zhao [4] studied the formation mechanism of aroma compounds in a glutathione–glucose reaction with fat or oxidized fat. Xu [5] revealed that, during thermal reactions of lipids, cysteine, and xylose, products 2-methylthiophene, 3-methylthiophene, 2-methyl-3-furanthiol, and 2-furanmethanethiol were the most crucial in aroma production in cooked meat and commercial meat flavors. In addition, many researchers have demonstrated an important stage in meat flavor production through pyrolysis or Strecker degradation in the participation of dicarbonyl compounds [6,7,8]. However, all of these studies pertained to the fat-mixing system involved in the reaction. Model systems such as cysteine-reducing sugar with or without a single lipid degradation product have scarcely been investigated.

Nevertheless, the origin of these compounds cannot be verified conclusively without labeling atoms with selectively enriched sugars and amino acids [9]. Therefore, the carbon module labeling (CAMOLA) approach is commonly used. It is a versatile and convenient tool for elucidating formation pathways of flavor compounds and for gaining information on the fragmentation of these precursors in a model reaction system or food matrix [10,11,12]. By the CAMOLA technique, especially 13C-labeled precursors, there are pathways to form cyclotenes, pyridinones, 2-acetyltetrahydropyridine, and several hydroxyproline-derived pyrrolyl and tetrahydroindolizinone compounds in [^1-13^C]glucose with proline and hydroxyproline systems, respectively [13], and those pathways are used to generate furans from an intact sugar skeleton [14] have been clarified. In addition, the formation pathways of 2-ethylthiophene, 2,5-dimethylthiophene, and 5-methylthiophene-2-carbaldehyde were explained in the glutathione and [^13^C_6_]glucose system [15].

In order to explore the role of aldehyde from lipid degradation in the formation of meat flavor compounds, a CAMOLA experiment was necessary. However, the cost of labeled aldehyde or amino acids is very high and their availability is also difficult. Thus, labeled sugar is usually used to indirectly elucidate the trend of aldehyde. The objective of this study was to elucidate the formation pathways of odorants formed from glucose, cysteine, and aldehyde during a Maillard reaction. The carbon module labeling approach is proposed to explain the extent to which the glucose skeleton remains intact during the formation of odorants and the extent to which breakdown products play a role. Additionally, creating a mixture of a carbohydrate (glucose) and an aldehyde (E-2-nonenal) with or without cysteine is proposed to elucidate the role of aldehyde in the formation of aroma compounds. In this approach, the sugar-containing carbon isomer labels (^13^C) and the peaks of selected volatiles were integrated in a total ion chromatogram (TIC) obtained from GC-MS, and the corresponding mass spectra were analyzed to assess the relative contribution of aldehyde to the formation of aroma compounds.

## 2. Materials and Methods

### 2.1. Materials

[^13^C_6_]Glucose (99%) was from Cambridge Isotope Laboratories, Inc. (Tewksbury, MA, USA). (E)-2-Nonenal, L-cysteine, and extra amounts of glucose were from Sinofarm Chemical Reagent Co., Ltd. (Shanghai, China). 1,2-Dichlorobenzene were of chromatography grade from TCI Development Co., Ltd. (Shanghai, China). C6–C30 n-alkane series (chromatographic reagent) were from Sigma-Aldrich Chemical Co. (St. Louis, MO, USA). 

### 2.2. Model Reactions

Cysteine, [^13^C_6_]glucose, and (E)-2-nonenal were dissolved in 10 mL of Na-phosphate buffer (0.2 mol/L, pH 6.00) and heated, combined with stirring in a 20 mL Maillard, septum-closed vial at 130 °C for 90 min in an oil bath. Similar model system experiments were also carried out according to conditions given in Table 1.

### 2.3. SPME(Solid Phase Microextraction)/GC-MS Analysis

All samples were analyzed three times with solid-phase microextraction fiber and assayed with a gas chromatography–mass spectrometer (SPME/GC-MS) and a carboxen/polydimethylsiloxane fiber. A 6 mL solution with 4 µL of a 1,2-dichlorobenzene methanol solution (100 mg/L) used as an internal standard was added into a 15 mL amber vial closed by a PTFE/silicone septum. 

Analysis of volatile compounds was performed on a GC-MS using an Agilent 5975C mass selective detector coupled with an Agilent 7890A GC (Agilent, Santa Clara, CA, USA), equipped with a DB-5MS capillary column (a 60 m × 0.25 mm inner diameter and a 0.25 µm film thickness). Helium was used as a carrier gas, and the flow rate was 0.8 mL/min. Subsequent headspace equilibration occurred over a period of 20 min at 60 °, and the SPME fiber was exposed in the headspace for 30 min. GC oven temperature was programmed at 40 °C (held for 3 min) and increased to 240 °C at 4 °C/min (held for 2 min). The MS conditions were as follows: the transfer line temperature was 280 °C; the ion source temperature was 230 °C; ionization energy was 70 eV, and mass range was 40–400 a.m.u. Desorption was in splitless mode at 250 °C for 5 min.

### 2.4. Qualitative and Quantitative Analysis of the Volatile Compounds

The volatile compounds were identified by comparing retention indices and retention times with those obtained for authentic standards or with mass spectra in the NIST 05 and Wiley 08 Database (Hewlett-Packard, Palo Alto, CA, USA). The retention indices (RIs) of unknown compounds were determined via sample injection with a homologous series of straight-chain alkanes (C6–C30) (Sigma-Aldrich, St. Louis, MO, USA).

Approximate quantities of the volatile compounds were estimated by comparison of their peak areas with that of the 1,2-dichlorobenzene internal standard, obtained from the total ion chromatograms, assuming that the relative response factor was 1 and the recovery ratio was 100%. The quantitative formula was as follows:

(1) $$W_{i}=f^{\prime }*\frac{A_{i}*m_{s}}{A_{s}}/m$$

where A_i_ is the peak area of compound i, A_s_ is the peak area of the internal standard, m_s_ is the mass of the internal standard, m is the mass of the sample, f′ is the relative correction factor, assumed to be 1, and W_i_ is the concentration (μg/g) of compound i.

### 2.5. Data Analysis

Data from the GC-MS analysis was evaluated by analysis of variance (ANOVA) using SPSS 21.0.

## 3. Results and Discussion

### 3.1. Volatile Compounds from Different Model Systems by GC-MS Analysis

The volatiles identified in the different model reactions are presented in Table 2. The results are mean values of three replicated experiments. The reaction between [^13^C_6_]glucose and (E)-2-nonenal (System A) resulted in the identification of 110 compounds. The main volatile compounds included 16 aldehydes, eight alcohols, seven furans, seven carboxylic acids, and others. The results showed that a high amount of (E)-2-nonenal was detected in the system, due to the fact that (E)-2-nonenal was not fully reacted in the reaction, which had arrived at the relatively high amount of 205.718 μg/g. The thermal reaction of (E)-2-nonenal produced degradation products such as hexanal, heptanal, octanal, and (E)-2-hexenal, as well as alcohols, ketones, acids, and esters. Therefore, the amounts of aldehydes, esters, acids, and alcohols of System A were much higher than those of Systems B and C, respectively. For example, the concentration of 2-nonenoic acid remained at 205.718 μg/g in System A, but was 0.066 μg/g in System B and 0.648 μg/g in System C. According to earlier research, (E)-2-nonenal can be converted to oxidized derivatives, such as 2-nonenoic acid, rapidly during malting in beer, even without amino acids [22]. In addition, because of the absence of amino acids, there were no sulfur-containing compounds or nitrogen-containing compounds detected in System A. 

In the reaction between [^13^C_6_]glucose and cysteine (System B), a total of 105 compounds were identified, including two aldehydes, two alcohols, six carboxylic acids, two furans, nine thiophenes, eight thiazoles, one imidazole, one pyrrole, and three thiols. These compounds correspond to many reports of reactions between cysteine and sugar in Maillard reactions [23]. Thiophene is an important sulfur-containing compound formed in Maillard reactions with cysteine, and it is a significant component in meat flavor [24]. Among the nine detected thiophenes, 4-butyl thiophene (0.078 μg/g) is attributed with a “floral, fruity, and milky” aroma, and dihydro-2-methyl-3(2H)-thiophenone (0.083 μg/g) emits a “sulfuric, fruity, and berry” note [25]. Meanwhile, two kinds of thiolsethanethiol and 2-thiophene thiol, important meat flavor compounds, have been detected [26]. It was found that the content of 2-thiophene thiol decreased to 0.80 μg/g when the reaction was supplemented with (E)-2-nonenal in System C; in System B, the content was 1.386 μg/g.

In System C, with [^13^C_6_]glucose, cysteine, and (E) -2-nonenal, 78 compounds were detected, including four aldehydes, six alcohols, six acids, three furans, six thiophenes, two thiazoles, one pyrroles, two pyridines, and three thiols. Due to the addition of (E)-2-nonenal in this system, 43 kinds of compounds were newly detected. Many researchers have reported that, when unsaturated aldehydes are added to a glucose and cysteine system, the types of volatile compounds change significantly, and the number of sulfur and nitrogen compounds are significantly reduced or even undetectable [27]. This might be because the aldehydes were added to the Maillard reaction to inhibit the formation of sulfur and nitrogen compounds in the reaction and to provide some small molecular weight carbonyl compounds so as to interact with and improve the odor of meat. It also can be seen in Table 2 that the addition of (E)-2-nonenal caused the disappearance of thiophenes such as dihydro-2-methyl-3(2H)-thiophenone, 2-butyl-thiophene, and thiophen. At the same time, new thiophene compounds, such as 2-pentylthiophene (0.779 μg/g), 3-hexylthiophene (0.098 μg/g), and 3-methyl-2-thiophenecarboxaldehyde (0.341 μg/g), were produced. Among them, 2-pentylthiophene has an odor of fatty attribute, and 3-methyl-2-thiophenecarboxaldehyde has an odor of camphoreous saffron. There are two kinds of pyridine compounds, of which 4-pentylpyridine (0.177 μg/g) has a fatty, green, and tallowy aroma and is an important contributor to meat flavor, which is why fat needs to be added to the preparation of meat flavors. 

### 3.2. The Origin of Carbons in Meat-Flavor Compounds from Different Models

Thermal reactions were carried out in 20 mL Maillard vials at 130 °C for 90 min and at a pH of 6.0. Due to the steam pressure at the reaction temperature, the customized, high-pressure-resistant, Maillard vials were used to avoid a possible breakage of glass. The quality of the vials was observed before and after the reaction, and no leakages were detected. The reaction was repeated with unlabeled glucose as control, and both reaction products were analyzed by SPME/GC-MS. Mass spectra of selected sulfur-containing and nitrogen-containing volatiles were analyzed.

#### 3.2.1. Reaction between [^13^C_6_]Glucose and (E)-2-Nonenal (System A)

Table 3 shows the isotope ratios of 2-pentylfuran and (E)-2-nonenal based on the molecular ions as generated by electron impact. A comparison of the isotope ratios of 2-pentylfuran revealed that the same isotopomer pattern occurred whether or not glucose (91.2%) or [^13^C_6_]glucose (89.1%) was used in the reaction. It can be seen in Table 4 that the ratio between unlabeled and fully labeled 2-pentylfuran was 89.2:10.8, indicating that either glucose or [^13^C_6_]glucose reacted with (E)-2-nonenal; a large proportion of 2-pentylfuran formed bears carbons from an unlabeled compound. The analysis of (E)-2-nonenal from the reaction in the presence of [^13^C_6_]glucose showed similar results. According to mass spectra, the main molecular ion isotopomers corresponded both to the unlabeled and the fully labeled compound (m/z 141). It shows that (E)-2-nonenal appears to mainly originate from its own addition. 

Table 4 gives an overview of the extent to which the C-skeleton of the selected volatiles stems from glucose (labeled carbon atoms) or from the degradation of (E)-2-nonenal (unlabeled carbon atoms). The compounds 2-furanmethanol, (E)-2-(1-pentenyl)-furan, and 2-pentylfuran were obviously derived from (E)-2-nonenal degradation because the unlabeled carbon atoms account for >90, >89, and 89%, respectively. It is also known that 2-pentylfuran belonging to the key odorants of the thermal Maillard reaction is a typical oxidation compound from linoleic acid and emits a “fruity and caramel malt” note. The odor activity values of 2-pentylfuran ranged from 201.8 in System A to 218.3 in System C. Because 2-pentylfuran has a relatively low threshold (0.006 µg/g), it could potentially play a crucial role in the aroma of Maillard reactions. It has been reported that the first hydroxylation at the allylic position and then cyclization could transform α,β-unsaturated aldehydes into 2-alkylfurans [1]. Both (E)-2-nonenal and glucose participated in the formation of 2-hexanoylfuran and (E)-2-nonenal, since a fraction of the carbon atoms was fully ^13^C_6_-labeled. 2-Hexanoylfuran represented a mixture of six isotopomers with a molecular mass of m/z 166 (64%), m/z 167 (11%), m/z 168 (4%), m/z 169 (7%), m/z 171 (4%), and m/z 172 (8%). It had just one unlabeled carbon left in the single incompletely labeled isotopomers. Thus, they were probably constructed by the incorporation of formaldehyde or acetaldehyde/mercaptoacetaldehyde, which came from the degradation of E-2-nonenal into an intact C-5 skeleton of glucose. The different isotopomers formally resulted from the possible combination of [^13^C_6_]glucose with (E)-2-nonenal, adding up to unlabeled, mostly 12.5% labeled 2-hexanoylfuran. (E)-2-Nonenal represented a mixture of three isotopomers with a molecular mass of m/z 140 (26%), m/z 141 (66%), and m/z 142 (8%). However, according to the result, (E)-2-nonenal was likely derived from the degradation of [^13^C_6_]glucose. Based on the above analysis, (E)-2-nonenal might have stemmed not only from the degradation of [^13^C_6_]glucose but also from its own residue. 

#### 3.2.2. Reaction between [^13^C_6_]Glucose and Cysteine (System B)

According to the literature, the sulfur-containing compounds ethanethiol, 5-methyl-2(5H)-thiophenone, 2-butyl-thiophene, and 1-methyl-5-mercaptotetrazole belong to the products of a heated solution of cysteine and sugar [28]. In the presence of cysteine, the detected furan derivatives declined significantly compared with System A. For example, 2-pentylfuran, described as a glucose/cysteine degradation product in the literature, was not detected in System B, possibly due to its low concentration and headspace analytical conditions [29]. From the five volatiles investigated in the reaction with labeled glucose in Table 5, compounds ethanethiol (>92%) and 1-methyl-5-mercaptotetrazole (>93%) were unlabeled; hence, the respective carbon atoms originated from cysteine. It was illustrated that 5-methyl-2(5H)-thiophenone was either unlabeled (42%, m/z 114) or fully labeled (34%, m/z 115; 12%, m/z 116; 7%, m/z 117). Potentially two different formation pathways exist with the integration of [^13^C_6_]glucose and cysteine carbons, respectively. These have been identified earlier as a cysteine degradation product and a reaction product from cysteine and glucose [30]. Instead, the carbon atoms of 2-butyl-thiophene and 5-butyldihydro-2(3H)-furanone mainly originated from the labeled compound. In accordance with previous reports, the 5-butyldihydro-2(3H)-furanone (No.7) and 2-butylthiophene (No.8) could result from the glucose-derived 3-deoxypentosone [31].

#### 3.2.3. Reaction between [^13^C_6_]Glucose, Cysteine and (E)-2-Nonenal (System C)

It can be seen in Table 6 that the four volatiles, which were investigated in the reaction with labeled glucose, were either unlabeled or fully labeled. Many different formation pathways probably exist with the integration of [^13^C_6_]glucose, cysteine, and (E)-2-nonenal. Table 7 shows the isotope ratios of 2-pentylthiophene, based on the molecular ions as generated by electron impact. A comparison of the isotope ratios of 2-pentylthiophene reveals that the proportion of reacted products formed with [^13^C_6_]glucose was equal to that with unlabeled glucose. In the reaction of unlabeled glucose, the mass of 2-pentylthiophene was m/z 154, accounting for 84.9%; similarly, in the reaction of [^13^C_6_]glucose, the proportion of 2-pentylthiophene was 86.7%. In Table 4, the formation of 2-pentylthiophene from the cysteine and α,β-unsaturated aldehydes is shown. Thiophene compounds are mainly produced by reactions of hydrogen sulfide and carbonyl compounds; in these reactions, 2-alkyl thiophene may be produced by hydrogen sulfide reacting with α,β-unsaturated aldehydes [32]. 

The 2-pentylfuran was mainly unlabeled (91%, m/z 138), and the isotope ratio was about 2% higher than that in System A. Meanwhile, the level of 2-pentylfuran was even higher in the [^13^C_6_]glucose, cysteine, and (E)-2-nonenal reaction mixture than in the [^13^C_6_]glucose and (E)-2-nonenal system. It illustrates that cysteine could promote the formation of 2-pentylfuran.

## 4. Conclusions

In this study, the key meat flavor compounds produced from the model Maillard reaction were successfully analyzed using a carbon module labeling technique. The results showed that, in the system of [^13^C_6_]glucose and (E)-2-nonenal, the main products of (E)-2-nonenal, 2-nonenic acid, and 2-pentylfuran were ^13^C_6_-labeled and were equally important for the formation of 2-pentylfuran, whether cysteine was present in the reaction or not. In the system of [^13^C_6_]glucose and cysteine and/or (E)-2-nonenal, the volatile compounds were significantly different when (E)-2-nonenal was added, and the detected sulfur and nitrogen compounds were significantly reduced in number or even undetectable. It was also found that the 2-furanmethanol, 2-hexanoylfuran, ethanethiol, (E)-2-(1-pentenyl)-furan, 5-methyl-2(5H)-thiophenone, 4-pentyl-pyridine, 1-methyl-5-mercaptotetrazole, 2-mercaptopropanoic acid, and 2-pentyl-thiophene were virtually ^13^C_1_–^13^C_4_ labeled, which suggests their origins. In addition, the resulting 5-butyldihydro-2(3H)-furanone and 2-butyl-thiophene were ^13^C_6_-labeled and hence stemmed from glucose. It is evident from this study that the relative contribution of lipid oxidation products to the C-skeleton of a particular aroma compound changed substantially when both glucose and cysteine were involved in the system. 

The use of ^13^C_6_-labeled glucose explained the extent of the fragmentation of the sugar skeleton. This study could be helpful for investigating the role of aldehydes as precursors in the generation of meat flavor compounds.

## Figures and Tables

**Table 1:** Model reactions ^a^.

	Amount					
	A	B	C	D	E	F
Cysteine	-	0.26 g	0.26 g	0.26g	-	0.26 g
[^13^C_6_]Glucose	0.13 g	0.13 g	0.13 g	-	-	-
Glucose	-	-	-	0.13	0.13 g	0.13 g
(E)-2-Nonenal	12.5 µL	-	12.5 µL	12.5 µL	12.5 µL	-

^a^ Reaction in phosphate buffer (0.2 mol/L; pH 6.00) at 130 °C (90 min).

**Table 2:** Volatile compounds identified in model systems.

	RI ^1^	Identification ^2^	Odors	Quantity (μg/g)		
				System A	System B	System C
**Aldehydes**						
Pentanal	<800	A,B,C	fruity, fermented, bready	0.232 ± 0.013 ^a^*	ND	ND
Hexanal	<800	A,B,C	fatty, green, grassy	0.849 ± 0.015 ^a^	ND	ND
(E)-2-Hexenal	867	A,B,C	green, banana, cheesy	20.986 ± 0.031 ^a^	ND	ND
(E,E)-2,4-Hexadienal	886	A,B	sweet, green, waxy	1.268 ± 0.05 ^a^	ND	ND
Heptanal	901	A,B,C	fatty, green, fresh aldehydic	7.166 ± 0.097 ^b^	ND	0.465 ± 0.016 ^a^
(E)-2-Heptenal	949	A,B,C	green, fatty	0.556 ± 0.017 ^a^	ND	ND
Benzaldehyde	968	A,B,C	bitter, oily, sweety	0.431 ± 0.015 ^a^	ND	ND
Octanal	1005	A,B,C	aldehydic, citrusy	8.632 ± 0.069 ^b^	0.079 ± 0.002 ^a^	0.133 ± 0.006 ^a^
(E)-2-Octenal	1081	A,B,C	fatty, green, waxy, cucumber	2.512 ± 0.094 ^a^	ND	ND
Nonanal	1104	A,B,C	fatty, rose, aldehydic	ND	0.088 ± 0.001 ^a^	0.319 ± 0.041 ^b^
(E)-2-Nonenal	1150	A,B,C	fatty, green cucumber	290.887 ± 0.044 ^b^	ND	0.556 ± 0.057 ^a^
2,4-Nonadienal	1176	A,B	fatty, green cucumber	7.017 ± 0.019 ^a^	ND	ND
(E,E)-2,6-Nonadienal	1266	A,B	fresh citrus, green cucumber	0.573 ± 0.018 ^a^	ND	ND
(E,E)-2,4-Decadienal	1275	A,B,C	oily, cucumber, melon	39.763 ± 0.036 ^a^	ND	ND
5-Hexyl-2-Furaldehyde	1430	A,B	-	0.522 ± 0.016 ^a^	ND	ND
4-Pentyl-Benzaldehyde	1448	A,B	-	1.516 ± 0.015 ^a^	ND	ND
2-Pentyl-2-Nonenal	1463	A,B	-	0.219 ± 0.067 ^a^	ND	ND
**Alcohols**						
2,3-Butanediol	<800	A,B	-	ND	ND	0.184 ± 0.02 ^a^
2-Butanol	<800	A,B	sweet, apricot	ND	ND	0.228 ± 0.034 ^a^
1-Pentanol	<800	A,B,C	fusel, oil, sweet	0.22 ± 0.013 ^a^	ND	ND
1-Hexanol	842	A,B,C	-	0.379 ± 0.013 ^a^	ND	ND
1-Heptanol	965	A,B,C	musty, leafy, violet	ND	ND	0.042 ± 0.001 ^a^
1-Octen-3-ol	991	A,B,C	mushroom, oily, green	1.007 ± 0.01 ^a^	ND	ND
2-Ethyl-1-Hexanol	1049	A,B	-	ND	0.077 ± 0.002 ^a^	ND
5-Methyl-6-Hepten-1-ol	1047	A,B	-	1.569 ± 0.043 ^a^	ND	ND
1-Octanol	1049	A,B,C	mushroom, rose, green	ND	0.634 ± 0.028 ^a^	ND
(Z)-2-Penten-1-ol	1094	A,B	green, plastic, fruity	0.224 ± 0.013 ^a^	ND	ND
(E)-3-Nonen-1-ol	1125	A,B	-	ND	ND	0.136 ± 0.006 ^a^
1-Butyl-Cyclohexanol		A,B	-	ND	ND	0.175 ± 0.004 ^a^
1-Methylcycloheptanol	1396	A,B	-	ND	ND	0.103 ± 0.004 ^a^
9-Decen-2-ol	1525	A,B	-	0.565 ± 0.038 ^a^	ND	ND
4-Cyclohexylidene-n-butanol	1721	A,B	-	0.153 ± 0.011 ^a^	ND	ND
5,7-Undecadienol	1826	A,B	-	0.23 ± 0.016 ^a^	ND	ND
**Acids**						
2-Propenoic acid	<800	A,B	-	ND	ND	0.091 ± 0.002 ^a^
3-Methyl-Butanoic acid	825	A,B	-	0.463 ± 0.019 ^a^	ND	ND
2-Methyl-2-Butenoic acid	883	A,B	-	ND	0.364 ± 0.029 ^a^	ND
Heptanoic acid	1078	A,B,C	sour, cheesy	6.72 ± 0.024 ^b^	ND	1.133 ± 0.012 ^a^
2-Ethyl-Hexanoic acid	1096	A,B	-	ND	0.077 ± 0.002 ^a^	ND
Octanoic Acid	1160	A,B,C	-	18.745 ± 0.034 ^c^	0.081 ± 0.002 ^a^	1.909 ± 0.021 ^b^
2-Octenoic acid		A,B,C	-	0.291 ± 0.046 ^a^	ND	ND
2-Methyl-Hexanoic acid	1204	A,B	-	0.325 ± 0.013 ^a^	ND	1.941 ± 0.028 ^b^
Nonanoic acid	1256	A,B,C	-	ND	1.904 ± 0.048 ^b^	0.611 ± 0.037 ^a^
2-Nonenoic acid	1310	A,B	musty, sour, oily, cardboard	205.718 ± 0.018 ^c^	0.066 ± 0.002 ^a^	0.648 ± 0.019 ^b^
n-Decanoic acid	1355	A,B	-	ND	0.087 ± 0.001 ^a^	ND
2-Methoxy-5-methylbenzoic acid	1534	A,B	-	0.423 ± 0.015 ^a^	ND	ND
**Furans**						
2-Furanmethanol	873	A,B	burnt, sweet, caramel	1.881 ± 0.013 ^a^	ND	ND
2-Pentyl-Furan	985	A,B,C	green, fruity, fatty	1.211 ± 0.024	ND	1.31 ± 0.021
2-Butyltetrahydro-Furan	1001	A,B	-	ND	0.076 ± 0.003 ^a^	ND
(E)-2-(1-pentenyl)-Furan	1060	A,B	phenolic coffee grounds	0.235 ± 0.018 ^a^	ND	ND
5-Hexyldihydro-4-methyl-2(3H)-furanone	1167	A,B	fruity, floral, lactonic, green	ND	ND	0.347 ± 0.029^a^
2-Hexanoylfuran	1283	A,B	sweet, green, beany	0.258 ± 0.01 ^a^	ND	ND
5-Butyldihydro-2(3H)-furanone	1218	A,B	-	ND	0.086 ± 0.001 ^a^	ND
4-Methylbenzofurazan	1393	A,B	-	ND	ND	0.093 ± 0.006 ^a^
5-Ethyldihydro-5-methyl-2(3H)-Furanone	1426	A,B	-	0.274 ± 0.026 ^a^	ND	ND
5-Methoxy-5-(acetoxymethyl)-2[5H]-furanone	1519	A,B	-	1.056 ± 0.033 ^a^	ND	ND
**Thiophenes**						
Thiophene	<800	A,B,C	-	ND	0.076 ± 0.003 ^a^	ND
Dihydro-2(3H)-thiophenone	952	A,B	burnt garlic	ND	0.082 ± 0.002	0.086 ± 0.003
Dihydro-2-methyl-3(2H)-Thiophenone	990	A,B	sulfur, fruity, berry	ND	0.083 ± 0.002 ^a^	ND
5-Methyl-2(5H)-thiophenone	1024	A,B	-	ND	0.081 ± 0.002 ^a^	ND
2-Butyl-Thiophene	1045	A,B	fruity, floral, milky	ND	0.078 ± 0.002 ^a^	ND
2-Pentyl-Thiophene	1160	A,B	fruity, fatty, cranberry	ND	ND	0.779 ± 0.012 ^a^
2(5H)-Thiophenone	1165	A,B	-	ND	0.083 ± 0.002 ^a^	ND
Thieno[3,2-b]thiophene	1192	A,B	-	ND	0.316 ± 0.021 ^a^	ND
3-Hexylthiophene	1244	A,B	-	ND	ND	0.098 ± 0.005 ^a^
2,2’-Bithiophene	1438	A,B	dry, phenolic	ND	3.415 ± 0.146 ^a^	10.734 ± 0.06 ^b^
1-(2-thienyl)-1-Heptanone	1575	A,B	-	ND	ND	0.064 ± 0.001 ^a^
5-(2-thienyl)-2-Acetylthiophene	1577	A,B	-	ND	0.076 ± 0.002 ^a^	ND
3-Methyl-2-thiophenecarboxaldehyde	1140	A,B	saffron (camphoreous)	ND	ND	0.341 ± 0.001 ^a^
**Pyridines**						
3-(2-Pyridyl)-1-propanol	1076	A,B	-	ND	ND	0.115 ± 0.003 ^a^
4-pentyl-Pyridine	1242	A,B	fatty, tallowy, green	ND	ND	0.177 ± 0.005 ^a^
**Thiazoles**						
5-methyl-Thiazole	825	A,B	-	ND	0.077 ± 0.003 ^a^	ND
tetrahydro-Thiazole	905	A,B	-	ND	ND	0.103 ± 0.004 ^a^
1,3,4-Thiadiazol-2-amine	991	A,B	-	ND	0.078 ± 0.003 ^a^	ND
1-Methyl-5-mercaptotetrazole	1071	A,B	-	ND	0.122 ± 0.003 ^a^	ND
Aminothiazole	1073	A,B	-	ND	0.173 ± 0.013	0.126 ± 0.001
4-methyl-2(3H)-Thiazolethione	1536	A,B	-	ND	0.075 ± 0.002 ^a^	ND
2-(2-methylpropyl)-thiazole	1479	A,B	-	ND	ND	0.086 ± 0.004 ^a^
2-Isobutylthiazole	1484	A,B	green, leafy, tomatoes	ND	0.117 ± 0.005 ^a^	ND
2-ethyl-2-methyl-Thiazolidine	1058	A,B	-	ND	0.078 ± 0.003 ^a^	ND
**Imidazoles**						
1-Imidazol-1-yl-3-methylbut-2-en-1-one	1123	A,B	-	ND	ND	0.106 ± 0.004 ^a^
2,5-dimethyl-1H-Benzimidazole	1295	A,B	-	ND	0.758 ± 0.013 ^a^	ND
**Pyrroles**			-			
1,5-dihydro-4-methoxy-2H-Pyrrol-2-one	1557	A,B	-	ND	0.079 ± 0.002 ^a^	ND
n-butyryloxy-2,5-Pyrrolidione	1606	A,B	-	ND	ND	0.703 ± 0.03 ^a^
**Thiols**						
2-Thiophenethiol	955	A,B	burnt, caramel, roasted coffee	ND	1.386 ± 0.042	0.926 ± 0.117
Ethanethiol	<800	A,B	sulfurous, fruity	ND	0.146 ± 0.006 ^a^	ND
1,2-Ethanedithiol	<800	A,B	-	ND	0.079 ± 0.002 ^a^	0.222 ± 0.015 ^b^
3-Methyl-4-amino-1,2,4-triazole-5-thiol	1186	A,B	-	ND	ND	0.086 ± 0.003 ^a^

^1^: RI, identified by retention indices (RI); ^2^: Method of identification: (A) mass spectrum comparison using NIST 08 and Wiley library; (B) mass spectrum and RI according to literature [16,17,18,19,20,21]; (C) mass spectrum and RI agree with that of the authentic compound run under the same conditions of the samples; ND: not detected in GC-MS; “-”: no volatile flavors; *. The values in the same line followed by different letters are significantly different (p < 0.05).

**Table 3:** Isotope ratios (%) of 2-pentylfuran and (E)-2-nonenal from the reaction of (E)-2-nonenal and glucose or [^13^C_6_]glucose, respectively.

2-Pentylfuran			(E)-2-Nonenal		
m/z	Glu	[^13^C_6_]Glu	m/z	Glu	[^13^C_6_]Glu
138	91.2	89.1	140	18.0	26.1
139	5.0	9.9	141	72.2	65.9
140	0.0	1.0	142	9.8	8.1
141	0.8	0.0	143	0.0	0.0
142	0.9	0.0	144	0.0	0.0
143	2.1	0.0	145	0.0	0.0
144	0.0	0.0	146	0.0	0.0

**Table 4:** Proportion of isotopomers formed from [^13^C_6_]glucose and (E)-2-nonenal.

No.	Compound ^a^	m/z (M+)	m/z (Analyzed Ions)	Unlabeled Carbon Atoms ^b^ (%)	Labeled Carbon Atoms ^b^ (%)	Number of Labeled Carbon Atoms
1	2-Furanmethanol	98	98;99	>90	<10	1
2	(E)-2-(1-pentenyl)-Furan	136	136;137	>89	<10	1
3	2-Hexanoylfuran	166	166;167;168;169;171;172	64	11:4:7:4:8	1;2;3;5;6
4	(E)-2-Nonenal	140	140;141;142	26	66;8	1;2
5	2-Pentylfuran	138	138; 139	89	11	1

^a^ Compounds were identified in the corresponding reaction between [^13^C_6_]glucose and (E)-2-nonenal by comparing the mass spectra and retention indices with those of authentic reference compounds, if not indicated otherwise; ^b^ Values represent the proportion between labeled and the indicated unlabeled isotopomers and are based on the abundance of the respective analyzed ion signals.

**Table 5:** Proportion of isotopomers formed from [^13^C_6_]glucose and cysteine.

No.	Compound ^a^	m/z (M+)	m/z (Analyzed Ions)	Unlabeled Carbon Atoms ^b^ (%)	Labeled Carbon Atoms ^b^ (%)	Number of Labeled Carbon Atoms
6	Ethanethiol	62	62:64	>92	<5	2
7	5-Methyl-2(5H)-Thiophenone	114	114;115;116;117	42	34;12;7	1;2;3
8	2-Butyl-Thiophene	139	139;140;141;142;143;144;145	19	13;16;12;12;14;13	1;2;3;4;5;6
9	1-Methyl-5-Mercaptotetrazole	116	116;117;118	>93	<7	1
10	5-Butyldihydro-2(3H)-Furanone	142	142;143;144;145;146;147;148	7	6;7;29;20;15;16	1;2;3;4;5;6

^a^ Compounds were identified in the corresponding reaction between [^13^C_6_]glucose and cysteine by comparing the mass spectra and retention indices with those of authentic reference compounds, if not indicated otherwise; ^b^ Values represent the proportion between labeled and the indicated unlabeled isotopomers and are based on the abundance of the respective analyzed ion signals.

**Table 6:** Proportion of isotopomers formed from [^13^C_6_]glucose, cysteine, and (E)-2-nonenal.

No.	Compound ^a^	m/z (M+)	m/z (Analyzed Ions)	Unlabeled Carbon Atoms ^b^ (%)	Labeled Carbon Atoms ^b^ (%)	Number of Labeled Carbon Atoms
11	4-Pentyl-Pyridine	149	149;150	>71	<26	1
12	2-Pentyl-Thiophene	154	154;155;156	87	9;4	1;2
13	2-Mercaptopropanoic acid	106	106;107;108	>89	4:4	1;2
5	2-Pentylfuran	138	138;139	91	9	1

^a^ Compounds were identified in the corresponding reaction between [^13^C_6_]glucose, cysteine, and (E)-2-nonenal by comparing the mass spectra and retention indices with those of authentic reference compounds, if not indicated otherwise; ^b^ Values represent the proportion between labeled and the indicated unlabeled isotopomers and are based on the abundance of the respective analyzed ion signals.

**Table 7:** Isotope ratios (%) of selected sulfur volatiles from the reaction of [^13^C_6_]glucose, cysteine, and (E)-2-nonenal.

2-Pentyl-Thiophene		
m/z	Glu	[^13^C_6_]Glu
154	84.9	86.7
155	7.7	8.6
156	6.1	4.2
157	1.0	0.4
158	0.1	0.1
159	0.1	0.1
160	0.1	0.00
